# Global Profiling of lncRNAs Expression Responsive to Allopolyploidization in *Cucumis*

**DOI:** 10.3390/genes11121500

**Published:** 2020-12-12

**Authors:** Panqiao Wang, Xiaqing Yu, Zaobing Zhu, Yufei Zhai, Qinzheng Zhao, Ya Meng, Ji Li, Qunfeng Lou, Jinfeng Chen

**Affiliations:** State Key Laboratory of Crop Genetics and Germplasm Enhancement, College of Horticulture, Nanjing Agricultural University, Nanjing 210095, China; 2016204024@njau.edu.cn (P.W.); xqyu@njau.edu.cn (X.Y.); 2016104051@njau.edu.cn (Z.Z.); zhaiyufei2012@163.com (Y.Z.); 2017204024@njau.edu.cn (Q.Z.); 2018104046@njau.edu.cn (Y.M.); liji1981@njau.edu.cn (J.L.); qflou@njau.edu.cn (Q.L.)

**Keywords:** lncRNAs, allopolyploid, *Cucumis*, transcriptome, interspecific hybridization

## Abstract

Long non-coding RNAs (lncRNAs) play critical regulatory roles in various biological processes. However, the presence of lncRNAs and how they function in plant polyploidy are still largely unknown. Hence, we examined the profile of lncRNAs in a nascent allotetraploid *Cucumis hytivus* (S_14_), its diploid parents, and the F_1_ hybrid, to reveal the function of lncRNAs in plant-interspecific hybridization and whole genome duplication. Results showed that 2206 lncRNAs evenly transcribed from all 19 chromosomes were identified in *C. hytivus*, 44.6% of which were from intergenic regions. Based on the expression trend in allopolyploidization, we found that a high proportion of lncRNAs (94.6%) showed up-regulated expression to varying degrees following hybridization. However, few lncRNAs (33, 2.1%) were non-additively expressed after genome duplication, suggesting the significant effect of hybridization on lncRNAs, rather than genome duplication. Furthermore, 253 cis-regulated target genes were predicted for these differentially expressed lncRNAs in S_14_, which mainly participated in chloroplast biological regulation (e.g., chlorophyll synthesis and light harvesting system). Overall, this study provides new insight into the function of lncRNAs during the processes of hybridization and polyploidization in plant evolution.

## 1. Introduction

Long noncoding RNAs (lncRNAs) are transcripts of more than 200 nucleotides in length that have no capacity to code for proteins [1]. LncRNAs typically originate from the intergenic regions of the genome and the antisense strands of coding genes [2]. Some lncRNAs can also be transcribed from the sense strands or intronic regions of coding genes [3]. In the past decade, thousands of lncRNAs have been widely identified in both animal and plant genomes because of rapid progress in high-throughput sequencing technologies [4,5,6,7,8,9]. In animals, increasing evidence supports that lncRNAs play critical roles in epigenetic regulation [10], for example, X chromosome inactivation [11,12], disease occurrence [13], genomic imprinting [14], developmental regulation [15], etc. In plants, lncRNAs are involved in many biological processes, including flowering time regulation [16,17], biotic and abiotic stimuli responses [18,19,20,21], photomorphogenesis [22,23], and regulation in other developmental processes [24,25]. In contrast to extensive studies of lncRNAs elucidated in mammals to date, the functions of plant lncRNAs are still obscure, especially in the process of polyploidization.

Interspecific hybridization and polyploidization are important in genome evolution [26]. Because the vigor traits created by hybridization are well stabilized during whole genome duplication (WGD), allopolyploids show their evolutionary advantages in development and adaptation [27,28,29]. The genomic interactions of two divergent subgenomes in a single nucleus trigger extensive and rapid genome restructuring, along with changes in DNA methylation, histone modification, and chromosomal rearrangements [30,31,32]. The combined results of these genomic and epigenetic changes lead to altered expression of about 20–50% of the mRNAs, providing a molecular basis for the viability of hybridization and polyploidization [3,33]. In nascent allohexaploid wheat, a high proportion of microRNAs showed nonadditive expression upon polyploidization [3]. However, the impact of hybridization and polyploidization on the other important types of noncoding RNAs are rarely reported [34].

The allotetraploid *Cucumis hytivus* (HHCC, 2n = 4x = 38) was synthesized through chromosome doubling of a hybrid between cultivated cucumber (*C. sativus*, CC, 2n = 2x = 14) and wild species *C. hystrix* (HH, 2n = 2x = 24) [35,36,37]. According to the molecular evidence from cytoplasmic and nuclear genomes, *C. hystrix* is classified as the closest wild relative of cucumber, diverging approximately 4.6 million years ago [38]. This *Cucumis* allotetraploid with a clear genetic background could facilitate the study of genetic, functional, and epigenetic changes during hybridization and genome duplication. Thus, transcriptomic analysis of synthetic allotetraploid *Cucumis* can shed light on the expression changes of transcripts following allopolyploidization. Previously, cDNA-AFLP and reverse Northern blot studies in early generations of *C. hytivus* (S_1_–S_2_) showed rapid activation or silencing of gene expression [39]. Recently, we have completed the high-quality assembly of *C. hytivus* genome, which make it possible to identify and characterize lncRNAs at whole-genome level in this newly synthetic allopolyploid specie in *Cucumis*. In this study, high-throughput RNA-seq was performed for genome-wide identification and characterization of lncRNAs in *C. hytivus*. To further investigate the potential roles of lncRNAs in interspecific hybridization and whole genome duplication in *Cucumis*, we examined the profile of lncRNAs in nascent allotetraploid *C. hytivus*, its progenitors and interspecific F_1_ hybrid. Study on the expression patterns of lncRNAs and their target genes will help us to better understand the epigenetic regulation mode of lncRNAs in allopolyploidization, and provide a molecular theoretical basis for distant hybrid breeding.

## 2. Materials and Methods 

### 2.1. Plant Materials and Growth Conditions

The following species were employed: cultivated cucumber *Cucumis. sativus* L. “BeijingJietou” (*C. sativus*, 2n = 14, genome CC), wild species *C. hystrix* (2n = 24, genome HH), and their interspecific F_1_ homoploid hybrid and synthetic allotetraploid *C. hytivus* (2n = 4x = 38, genome HHCC). The highly inbred synthetic allotetraploid *C. hytivus* (S_14_) were obtained by self-pollinating 14 generations from the original chromosome doubling of interspecific F_1_ (designated as S_0_) (Figure S1a,b). All planting materials were supplied from the state key laboratory of Cucurbit Genetics and Germplasm Enhancement of Nanjing Agricultural College. Seeds of five samples were sown on plastic pots and grown under controlled conditions (14 h light and temperature range of 22–28°C), with a relative humidity of 70–80%.

### 2.2. RNA Isolation, cDNA Library Preparation, and Illumina Sequencing

The first young leaf of each seedling with three biological replicates were collected at ten-leaf stage, immediately frozen in liquid nitrogen, and then stored at −80˚C for further use. The extraction of total RNA and synthesis of first-strand cDNA were performed using Trizol reagent (TaKaRa Bio Inc., Kusat su, Shiga, Japan) and the PrimeScript TM RTPCR kit (TaKaRa) according to the manufacturer’s instructions, respectively.

After RNA extraction, the purity, concentration, and integrity of RNA were tested using NanoDrop 2000 (Thermo Fisher Scientific, Wilmington, DE, USA). The poly(A)^+^-type cDNA libraries for the leaf samples collected from different species were constructed and subjected to high-throughput sequencing provided by 1 Gene Ltd. (Hangzhou, China). The libraries were sequenced on Illumina HiSeqTM 2500/MiSeq platform with 150-bp paired-end reads. The datasets generated during the current study are available in the National Center for Biotechnology Information (NCBI) under the bioproject accession number PRJNA658669.

### 2.3. Transcriptome Assembling and Identification of lncRNAs 

Raw reads obtained from RNA-Seq were pre-processed. The 5′ and 3′ adapters were trimmed; low-quality (Phred Score ≤ 20) and shorter reads (length ≤ 30 bp) were removed by FASTX-toolkit v0.0.13 (http://hannonlab.cshl.edu/fastx_toolkit/). The clean reads were mapped to the reference genome (S_14_ genome assembled in our lab using SMRT sequencing technology, Cucumis Genome Database: http://www.cucumisgdb.cn/) using Hisat2 v2.1.0 (-p 20 –dta) [40], and were assembled using StringTie (v1.3.4d) [41]. The length of all transcripts should be no less than 200 bp. The assembled transcripts were annotated using the Cuffcompare program from the Cufflinks package (v2.2.1) (http://cufflinks.cbcb.umd.edu/). According to Cuffcompare, only transcripts class-coded as “u” (unknown intergenic transcript), “i” (a transfrag falling entirely within a reference intron), and “x” (exonic overlap with reference on the opposite strand) were selected as novel transcripts including long non-coding RNA (lncRNA).

Then, the candidate lncRNA was identified based on the coding potentials score, which was calculated by using the coding potential calculator (CPC2) [42], PLEK [43], CPAT [44], and CNCI [45]. The intersection with CPC2 score < 0, PLEK score < 0, CNCI score < 0, and CPAT score < 0.36 were the initial predicted lncRNAs [42,43,44,45]. The Venn diagram of the lncRNA list from each software were prepared using the function vennDiagram in R. The predicted lncRNAs with at least two exons were used for the next analysis. The lncRNAs were classified into several categories based on their genomic location, according to description in previous studies [46]. RepeatModeler v.1.0.11 (-LTRStruct -engine ncbi -pa 18) (http://www.repeatmasker.org/RepeatModeler.html) was used to construct de-novo transposable element (TE) libraries based on the *C. hytivus* reference genome using default parameters. The initial repeat database was then merged with the known Repbase database (20170127), which was used to distinguish the *C. hytivus* TEs by using RepeatMasker v.4.0.9 (-parallel 18 -engine ncbi -nolow -no_is -norna -gff) (http://www.repeatmasker.org). TE-derived lncRNAs were identified by the determining overlapping genomic coordinates of TEs or TE fragments of at least 10 bp using the intersect program from BEDTools v.2.17.0 (https://github.com/arq5x/bedtools2) with reference to previous studies [34,47]. The comparative analysis of syntenic orthologous gene and lncRNA pairs were performed using blast (blastn -evalue 1e-5 -max_target_seq 1) and MCScanX (-b 2, -s 4) [48]. The co-linear pairs were determined by one to one optimal matching results from BLASTn. The longest transcripts (mRNA) of 46,845 protein coding genes (PCGs) annotated in the *C. hytivus* reference genome were used to comparing sequence characteristics of lncRNA. The sequence characteristics of the lncRNAs were visualized in ggplot2 R-package (v3.1.0) and Circos v0.69-3 [49].

### 2.4. Differential Expression Analysis

Expression levels of genes and lncRNAs were measured with normalized counts of reads by their respective lengths using kallisto v0.44.0 (quant –t 20 –b 100) [50]. Then we collapsed the expression of lncRNA into gene level by an in-house R script. Transcripts per million mapped reads (TPM) was applied to represent the normalized expression value. Heatmap of samples was generated using the R package pheatmap v1.0.10 (https://cran.r-project.org) and the Pearson correlation between biological replicates was calculated using the function cor in R based on the TPM values. The average values of the two parents’ expression levels (reads count and TPM) were calculated as mid-parent value (MPV), which were used for subsequent analysis. The expression profiles of lncRNAs were clustered and visualized by Java program Short Time-series Expression Miner (STEM v1.3.11) [51] under the default parameters. DEseq2 R package (v1.20.0) was used to estimate variance-mean dependence in count data from kallisto and test for differential expression based on a model using the negative binomial distribution [52]. Because lncRNAs exhibited more expression variance than PCGs, lncRNAs with a p-adjust < 0.0001 (Benjamini-Hochberg [BH] multiple test correction) and log_2_(fold change) >2 or <−2 were set as the threshold of the significantly differential expression [34,53]. The species specific expression was classified as a type of differential expression.

### 2.5. Prediction of lncRNA Function

The *cis*-acting mode of lncRNA is the main regulating function on their neighboring genes [54,55]. Thus, the protein-coding genes in 100k upstream and downstream of lncRNA were searched and predicted as potential target genes (PTGs). A Pearson correlation of the expression of lncRNA and its target gene pair was calculated using the function cor in R. Protein-coding gene function was annotated using BLAST 2.6.0+ (blastp -evalue 1e-6 -outfmt 7 -num_threads 24) (https://blast.ncbi.nlm.nih.gov/Blast.cgi), Gene Ontology (GO) classify (blast2go.jar -in alignment_result.xml -a -v) (http://geneontology.org/) and in-house R scripts based on the following databases: NCBI Non-Redundant Protein Sequences Database, Swiss-Prot for a manually annotated and reviewed protein sequence database and GO. GO enrichment analysis of the target genes for differentially expressed (DE) lncRNA was conducted by clusterProfiler R package (v3.16.0) [56]. 

### 2.6. qRT-PCR Analysis for DE lncRNAs and Their Targets

The relative quantity of lncRNAs and their targets were evaluated by Real-time fluorescent quantitative polymerase chain reaction (qRT-PCR). All primer sequences were designed by software Primer Premier 6.0 software and listed in Table S1. qRT-PCRs were performed with the SYBR Premix Ex TaqTM II kit (TaKaRa) and CFX96 real-time detection system (Bio-Rad Laboratories Inc., Hercules, CA, USA) according to the manufacturer’s protocol. Three biological replicates and three technical replicates were assigned. The actin gene was used as an internal control for data normalization, and quantitative changes in different replicates were calculated using the delta-delta threshold cycle relative quantification method according to Yu et al. [57]. Significant differences were determined by Duncan’s multiple range test at *P* = 0.01 level in SPSS. The fitting and visualization of the regression curve for qPCR and RNA-seq quantitative results were performed in R 3.4.1 (http://www.r-project.org/).

### 2.7. Photosynthetic Parameters Measurements

The net photosynthetic rates (Pn) and stomatal conductance (Gs) were measured using a Li-6400 portable photosynthesis assay apparatus (LI-COR Biosciences Inc., Lincoln, NE, USA). The leaf photo-synthetically active radiation (PAR) was controlled at 12 levels from 0 to 1500 μmol m^−2^ s^−1^ (0, 25, 50, 75, 100, 150, 200, 300, 600, 900, 1200, and 1500). The maximum net photosynthesis rate under saturation light (Pmax) was obtained by using Photosynthesis Work Bench (Li-Cor, Lincoln, NE, USA) according to the method of Yu et al. [58].

## 3. Results

### 3.1. Identification of the Putative lncRNAs in C. hytivus Leaves

**Figure 1:**
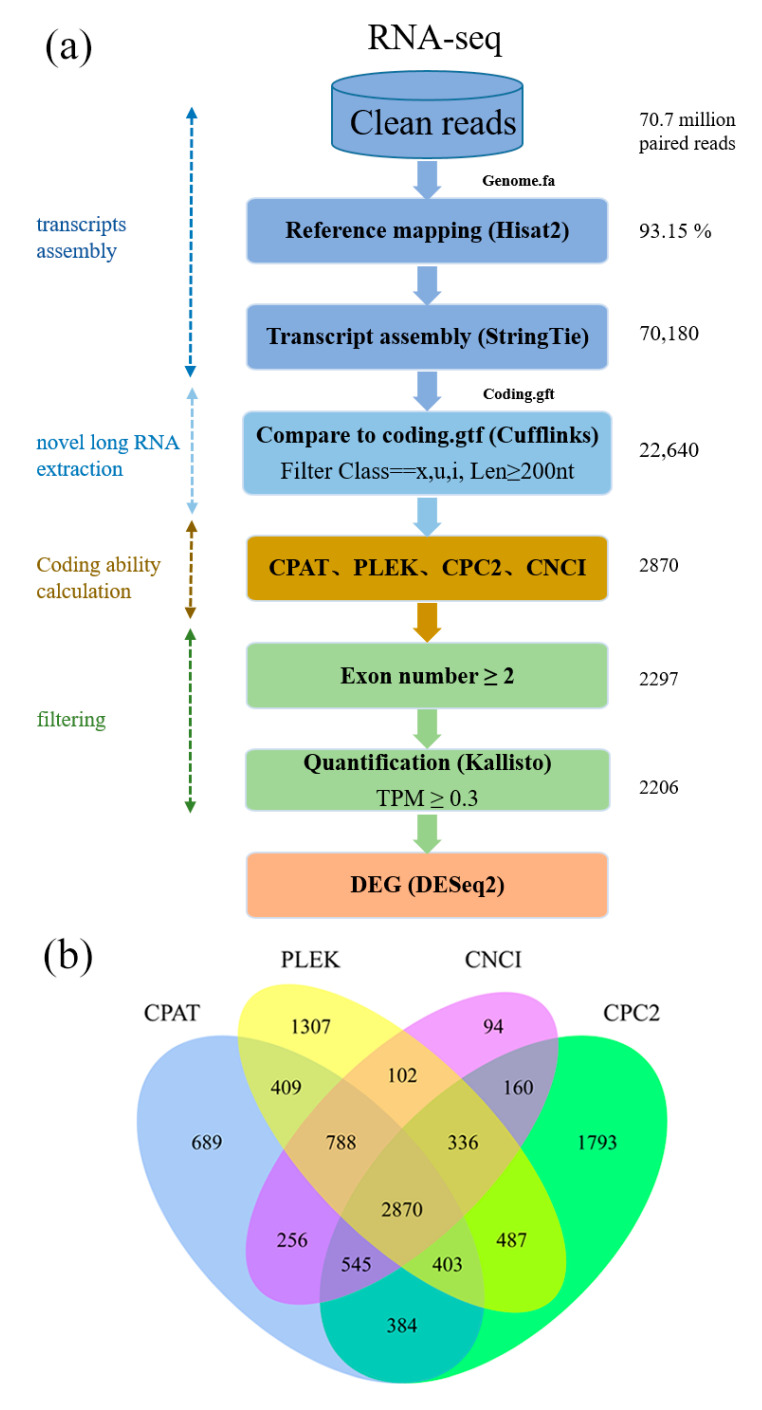
Complete computational pipeline for the systematic identification of long non-coding RNA (lncRNAs) in allotetraploid *Cucumis hytivus* leaves. (**a**) Detailed schematic diagram of the bioinformatic pipeline for the identification of leaf lncRNAs. (**b**) Venn diagram of the number of candidate lncRNAs filtered by CPC2, CNCI, CPAT, and PLEK. CPC2: coding potential calculator; CNCI: coding-noncoding index; CPAT: coding potential assessment tool; PLEK: a tool for predicting long non-coding RNAs and messenger RNAs based on an improved *k-mer* scheme.

To identify lncRNAs in *C. hytivus*, we established a pipeline (https://github.com/wangpanqiao/lncRNA_Pipeline) consisting of four main steps: transcript assembly, novel long RNA extraction, protein-coding capacity prediction, and filtering, as shown in Figure 1a. We first obtained 70,701,732 clean reads from the library in leaves of *C. hytivus* (S_14_), with 93.15% of which were successfully mapped to reference genome. Then, mapped data were used to assemble transcripts by StringTie. In total, 70,180 assembled transcripts were obtained. Assembled annotation file gtf was compared with the reference to distinguish novel long RNAs by filtering transcripts location not overlapping with the known gene and excluding length smaller than 200 nt. As a result, 22,640 transcripts were obtained after extracting. Then, 2870 transcripts without coding ability were singled out from the intersection of the CPC2, CNCI, CPAT, and PLEK results (Figure 1b). Finally, these non-coding potential transcripts were retained under two criteria: (1) exon number >= 2; (2) TPM > 0.3, and were defined as credible lncRNA with total 2206 transcripts transcribed from 1593 unigenes (lncRNA-gene). CPAT was used to test the coding probability of final discriminated lncRNAs and protein coding transcripts, and almost all of the lncRNAs were less than 0.36 (Figure S2).

### 3.2. Sequence Characteristics of the lncRNAs

**Figure 2:**
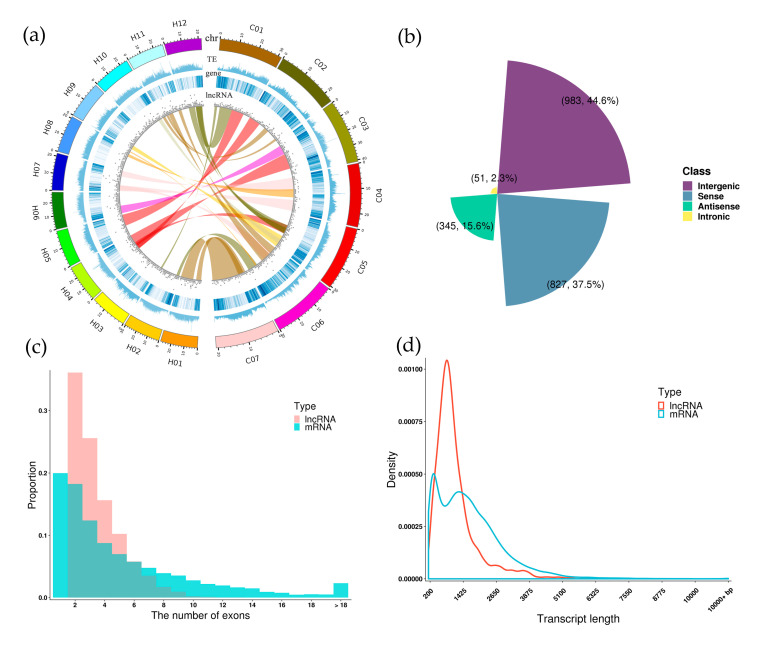
Description of the sequence characteristics of lncRNAs in *C. hytivus* leaves. (**a**) Analysis of the lncRNAs along each chromosome, showing density of genes, transposon elements, and lncRNAs in physical bins of 100 kb for each chromosome. The circles from inside to outside represent lncRNA, gene, transposon elements (TE), and chromosomes (chr). The internal collinear bands represent 145 lncRNAs in 23 synteny blocks. The scale for the chromosomes (outer bars) is Mb. (**b**) Numbers of intergenic, intronic, antisense, and sense lncRNAs in *C*. *hytivus* leaves. (**c**) Numbers of exons of lncRNAs in comparison with protein-coding transcripts of *C*. *hytivus*. (**d**) Comparison of the transcript length distributions of lncRNAs and mRNAs.

To gain a comprehensive understanding of the distribution and structure of lncRNAs in *C. hytivus*, we then investigated their exon numbers, transcript length, and overlapping with transposable elements (TEs). The lncRNAs that could be assigned to a chromosome location were more evenly distributed across the 19 chromosomes than TE and PCGs in *C*. *hytivus* with no obvious preferences of locations. The number of lncRNAs in two subgenomes were 1406 (subH) and 675 (subC) respectively (Figure 2a). The remaining 125 lncRNAs were located on the scaffold fragments. Between the two subgenomes, 145 pairs of lncRNA-genes showed good collinearity, which was consistent with the collinearity of the coding genes (Figure 2a; Figure S3a,b). Especially, H01 and C07 maintained good collinearity in two subgenomes. However, some lncRNAs from the chromosome C05 of the subC genome showed better collinearity with the lncRNAs on the chromosomes H12 and H04 of the subH genome, which was absent in the collinearity of the encoding genes in two subgenomes (Figure S3a,b).

Among these lncRNAs, 983 intergenic (lincRNAs), 827 sense, 345 antisense, and 51 intronic lncRNAs were identified according to the locations of lncRNAs in the genome (Figure 2b). With respect to exon numbers, we found that the range of exon numbers in lncRNA transcript was more concentrated than mRNA, and most of the lncRNAs (61.7%) contained two or three exons (Figure 2c). Only a few intergenic lncRNAs (1.9%) and sense lncRNAs (1.5%) contained more than nine exons (Figure S4a). The mean transcript length (1016 bp) of the lncRNAs was obviously shorter than that of protein-coding genes (1490 bp). Most lncRNAs (84.1%) had length shorter than 1500 nucleotides (Figure 2d). The majority of the antisense lncRNAs (85.5%) were shorter than 1000 nucleotides (Figure S4b). When using ≥10 bp as an overlapping criterion, the proportion of lncRNAs overlapping with TEs (76.7%) was much more than that of mRNAs (51.2%) (Table S2) possibly because of the less conservativeness of ncRNAs than coding genes.

### 3.3. Profiling of lncRNA Expression in Interspecific Hybridization and Genome Duplication

**Figure 3:**
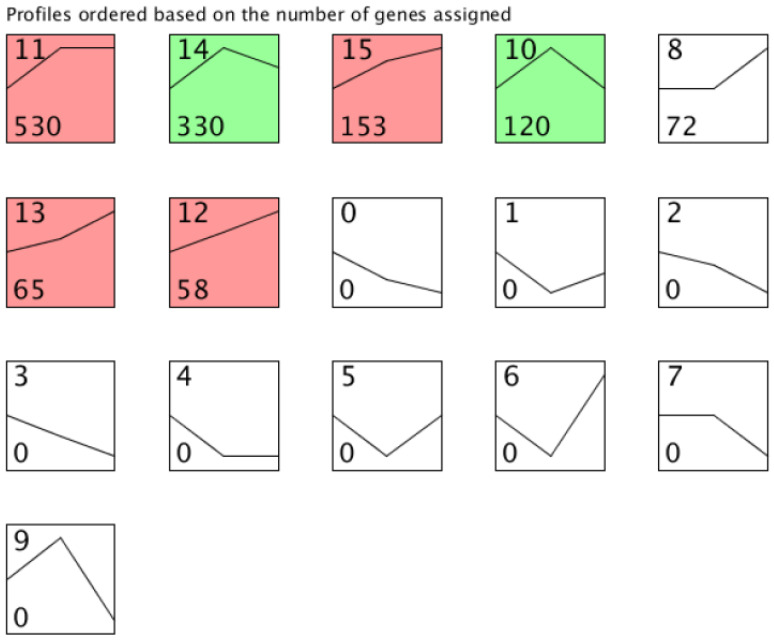
Expression profile clusters of lncRNAs across MPV, F_1_, allotetraploid S_14_. The number in the top left-hand corner of a profile box is the profile ID number; bottom number represent number of genes assigned to the expression profile. The colored profiles had a statistically significant number of genes assigned. Non-white profiles of the same color represent profiles grouped into a single cluster. MPV: mid-parent value.

To identify the dynamical model of lncRNA expression in three different stages (hybridization, duplication, and diploidization), we constructed four RNA-seq libraries from leaves of two diploid parents (CC, HH), their interspecific F_1_ hybrid, and synthetic allotetraploid (S_14_) for high-throughput sequencing (Figure S1a–c). From CC, HH, and F_1_, we respectively obtained 72,823,501, 72,036,788, and 70,755,534 clean reads. For interlibrary comparison, read numbers were normalized to relative abundance transcripts per million mapped base pairs (TPM) [50]. Gene expression correlations between three biological replicates were high, with average Pearson correlation coefficients (lncRNAs and mRNAs) of 0.99, 0.98, 0.98, and 0.98 for CC, F_1_, HH, and S_14_, respectively (Figure S5a,b). In stark contrast to PCGs, we found that the expression level of lncRNAs (log_2_TPM) exhibited much lower than mRNAs across the four samples (Figure S6; Table S3). The lncRNAs expression levels of hybrid F_1_ and synthetic tetraploid were between two diploid ancestors (Table S3). To identify significant temporal expression profiles across allopolyploidization process, 1593 lncRNA-genes expression data from two progenitors, hybrid F_1_ and S_14_ progeny were clustered in the Java program STEM. The expression of lncRNAs changed regularly along with allopolyploidization. Here, lncRNAs were classified into seven bins (as shown in Figure 3) according to their expression patterns among the mid-parent value (MPV) and the progeny (MPV-F_1_-S_14_). We found that 1328 lncRNA-genes showed varying degrees of change during the allopolyploidization, of which 1256 (94.6%) were activated during interspecific hybridization. Following, there were 348 lncRNA-genes increased and 450 lncRNA-genes decreased during polyploidization, respectively (Figure 3; Table S4). After genome duplication, up-regulated lncRNA accounted for 66.1% (excluding expression profile 10 and 14). The expression profile 11 contained the most genes (530), which were up-regulated in F_1_ and maintained the similar expression level in S_14_. These results suggested that hybridization may have caused more lncRNAs of up-regulation. 

### 3.4. Different Expression of lncRNA during Cucumis Allopolyploidization

**Figure 4:**
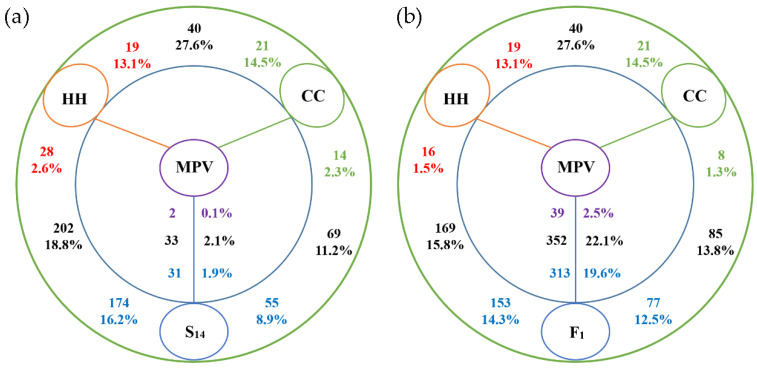
lncRNA genes differentially expressed in S_14_ (**a**)/F_1_ (**b**) progeny and their diploid (HH, CC) progenitors. Numbers close to the species (colored) represent upregulated lncRNAs compared with the neighboring species. Percentages indicate those among all expressed lncRNAs. The total number of lncRNAs differentially expressed between the two species is given (black).

To study the effect of allopolyploidization on lncRNA expression in the *Cucumis* synthetic allopolyploid, we first compared lncRNA expression levels between the two progenitors. Among 145 collinear lncRNA-genes expressed in leaves, 27.6% (40) differentially expressed (*p* < 0.0001) between *C. hystrix* and *C. sativus* parental lines (HH-CC_dif_) (Figure 4a; Figure S7a). Furthermore, 19 (13.1%) were expressed at a higher level in the wild species (HH > CC), with the remaining more highly expressed in the cultivated cucumber (HH < CC, 14.5%, 21). 

The differential expression of lncRNAs between the S_14_ and each parental line was then studied. There were 202 (18.8%) differentially expressed lncRNAs (DE-lncRNAs) between *C. hytivus* S_14_ and *C. hystrix* (S_14_-HH_dif_) which is more than comparison between S_14_ and *C. sativus* (S_14_-CC_dif_). In S_14_-CC_dif_ lncRNAs, 55 lncRNA-genes expressed higher in S_14_, accounting for 8.9% of the total expressed lncRNA-genes (617, subC). It was more than the ratio (2.3%, 14) of lncRNAs highly expressed in parental *C. sativus* (Figure 4a). Similar patterns were found for S_14_-HH_dif_ (Figure 4a and Figure S7c), the number of higher expressed lncRNAs was smaller in progenitor HH (28; 2.6%) than S_14_ (174; 16.2% of the total 1073 subH lncRNA-genes). However, the difference ratio between S_14_ and female parent (HH; 18.8%) was larger than the male parent (CC; 12.2%), indicating that allotetraploid *Cucumis* is indeed more highly diverged from *C. sativus* than from *C. hystrix* in terms of lncRNAs transcriptome profiles. To determine the non-additively expressed lncRNAs, the average of parental lncRNAs expression levels (mid-parent value; MPV) were compared with nascent allotetraploid progeny S_14_. Results showed that non-additively expressed lncRNAs-genes (*p* < 0.0001) only represented a small percentage (2.1%; 33) of the total lncRNAs.

In order to distinguish the effects of hybridization and genome doubling on lncRNA expression, the interspecific F_1_ hybrid was used to compare with its parents and MPV. The results of differential expression analysis showed that the lncRNAs expressed higher in F_1_ were 9.56 (153:16) and 9.62 (77:8) times more than that in HH and CC, respectively (Figure 4b). Contrary to the result in allotetraploid progeny S_14_, a high portion (22.1%, 352) of the expressed genes were identified as nonadditively expressed lncRNA genes in F_1_ when compared with MPV derived from the two parental lines. 88.9% (313) of these non-additively expressed lncRNA-genes (MPV-F_1dif_) were highly expressed in interspecific F_1_ hybrid progeny (Figure 4b), of which 65 lncRNA-genes were highly expressed in F_1_ hybrid when compared with its parents (F_1_-CC_dif_, F_1_-HH_dif_) (Figure S8). Of the differentially expressed lncRNA-genes between parents, only 2 (10.5% of HH highly expressed; 19) and 1 (4.8% in CC highly expressed; 21) lncRNAs were retained in the F_1_ hybrid (Figure S8). The number of non-additively expressed genes in F_1_ to S_14_ reduced from 352 to 33. Among 352 non-additively expressed genes, only three lncRNA-genes (LINC-chh12G002100-1, LINC-chh10G005690-1, LINC-chc05G015470-1) were transgressively heritable across F_1_ and S_14_ generations (Figure S9). After interspecific hybridization, a total of 148 lncRNA-genes showed significantly differential expression in leaves of *C. hytivus* S_14_ compared with interspecific F_1_ hybrid, including 69 upregulated genes and 79 downregulated genes (Figure S7f).

Overall, these results indicated that the expression of lncRNAs were up-regulated after the distant hybridization was completed. Then, most of the lncRNAs remained stable in polyploidization, and another batch of lncRNAs had up-regulated or down-regulated differentiation. Moreover, the number and difference in polyploidization were much lower than in the hybridization process, suggesting these allopolyploidy-related lncRNAs might play different roles in the two important stages.

### 3.5. Functional Analysis of Allopolyploidization-Related lncRNAs

**Figure 5:**
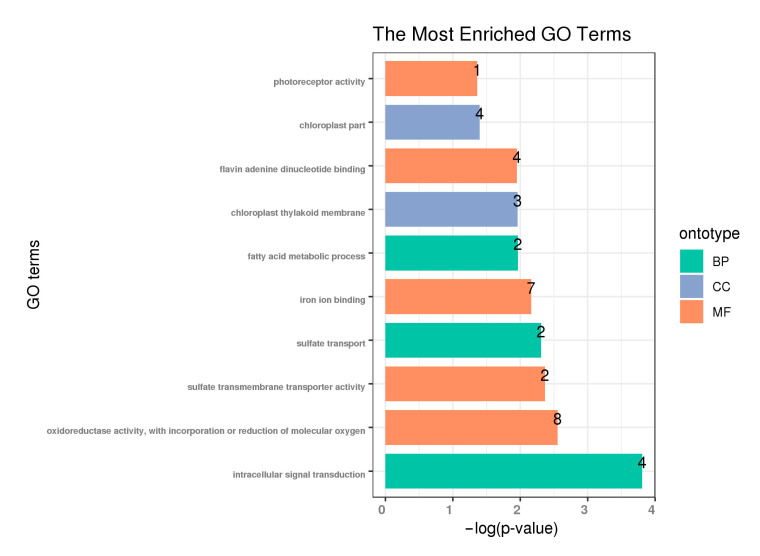
Gene Ontology (GO) enrichment analysis of potential target genes (PTGs) of differently expressed lncRNAs in allotetraploid S14 compared with parents/MPV. Shown are significantly enriched GO terms (top10, *p*-value < 0.05). The label at the top of the bar represents the number of genes. BP, biological process; MF, molecular function; CC, cellular component.

To reveal the potential functions of the allopolyploidization-related lncRNAs, the union of DE-lncRNAs between *C. hytivus* S_14_ and its diploid ancestors (or MPV) was obtained. We predicted 253 genomic co-location protein-coding (*cis*-regulated) genes as potential target genes (PTGs) of the DE-lncRNAs to participate in allopolyploidization. Gene Ontology (GO) functional categories of PTGs revealed enrichment for photosynthesis (Figure 5; *p*-value < 0.05), including four chloroplast cellular component genes (GO:0044434) in the cellular components category, seven iron-ion-binding genes (GO:0005506) and eight oxidoreductase activity genes (GO:0016705) in the molecular function category. In addition, there were two PTGs involved in sulfate transport and fatty acid metabolism, respectively.

Hybridization and polyploidization are two crucial processes in genome evolution. To exploit more possible functions of the hybridization-related lncRNAs, the union of DE-lncRNAs between interspecific F_1_ hybrid and its diploid ancestors (or MPV) was obtained. We predicted 501 *cis*-regulated protein-coding genes as PTGs of the DE-lncRNAs to participate in interspecific hybridization. GO enrichment analysis of PTGs revealed enrichment for translation function (Figure S10; *p*-value < 0.05), including 15 genes related to structural constituent of ribosome (such as ribonucleoprotein complex) and seven constituent genes of mitochondria. Furthermore, target genes of DE-lncRNAs also correspond to chloroplast composition and photosynthesis-related processes, which are mainly involved in stomatal complex development (two), chloroplast organization (nine), light-harvesting (two), chlorophyll biosynthetic process (two). 

The results of functional enrichment of target genes suggested that allopolyploidization-related lncRNAs (differentially expressed in allopolyploidy) plays an important role in the regulation of biological processes of organelles, especially in photosynthesis. Among these lncRNAs, abundance expression of 43 lncRNAs related to chloroplast were visualized in heatmap (Figure S11; Table S5). The absolute value of correlation coefficient between lncRNAs abundance and corresponding PTGs ranged from 0.62 to 0.99 according to *p*-value at 5% level, of which chc03G019620-INside-1, chc03G030700-AS-1, chc06G011860-AS-1, chh06G012250-INside-1, chh10G006290-AS-1, chh10G012720-AS-1 and their candidate target genes reached 0.99 (Table S6). These results implied the possibility of molecular connection among lncRNAs and PTGs induced by polyploidization. Given the variation of leaf color of hybrid and allotetraploid, the chlorophyll content was measured in each species. As shown in our previous findings, the leaf chlorophyll (Chl) content of *C. sativus* (CC), *C. hytivus* (HHCC), and *C. hystrix* (HH) were 19.67, 15.38, and 29.06, respectively [58]. The target gene of LINC-chc01G00070-1 down-regulated in *C. hytivus* (log_2_FC: −1.63) was the key gene (*CHLM*) in chlorophyll biosynthetic process, which was also downregulated (log_2_FC: −1.60; Table S6) when compared with the paternal parent (CC). In order to study the final effect of polyploidy on chloroplast activity and photosynthesis, photosynthetic parameters were measured in each species. The net photosynthetic rates (Pn) in *C. hytivus* were in the middle level between parents with the light change (0–1500 μmol m^−2^ s^−1^) (Figure S12a). The stomatal conductance (Gs) of *C. hytivus* and *C. hystrix* was lower than that of *C. sativus*, even when the light intensity was increased (Figure S12b). Initially the maximum net photosynthesis rate (Pmax) of *C. hytivus* was similar to that of *C. sativus* but lower than in *C. hystrix* (Figure S12c). However, the maximum quantum efficiency of photosystem II (F_v_/F_m_) of *C. × hytivus* showed over-parent genetics [58], suggesting that the photosystem II might be regulated by lncRNA associated with polyploidy. Similar to *CHLM*, lncRNAs (chc06G011860-AS-1, chc05G021510-AS-1) and their target genes, chlorophyll-binding protein gene (*LHCP*), were downregulated in S_14_, and the target gene was downregulated by 2.46 and 2.36 times respectively. It was also found that the DE-lncRNAs were associated with the changes of key genes in photorespiration (*CRR6*), stomatal complex development (*MEPF2*, *MEPFL3*), and carbon fixation (*RuBisCO* small chain) (Tables S5 and S6). The allotetraploid maintained higher level of photosynthesis may be through improving the thylakoid membrane lipid components and function of protein complexes under genome shock.

### 3.6. qPCR Verification for DE-lncRNAs and Their Target Genes 

**Figure 6:**
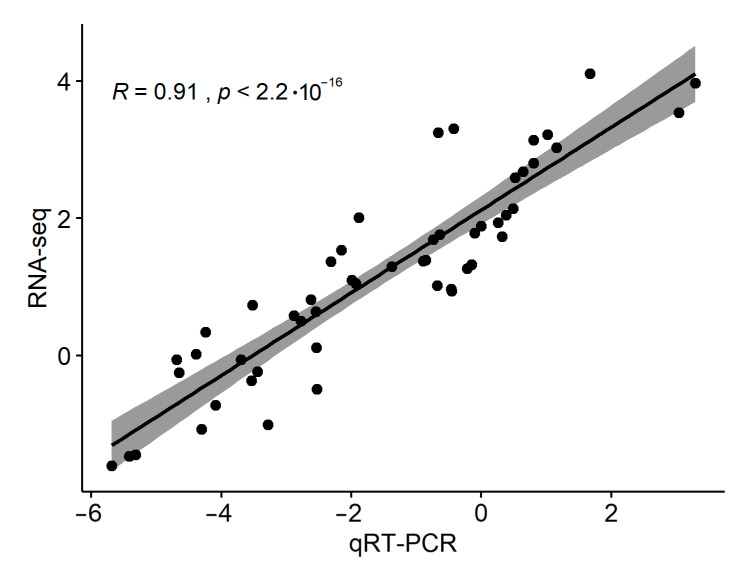
Transcript abundance estimated from qPCR analysis and from RNA-Seq data. The *y* axis represents log_10_(TPM), and the *x* axis represents log_10_(2^−ΔCt^). The shadow band shows the confidence interval.

To confirm the validity of RNA-seq data, 14 differentially expressed genes (including 8 lncRNAs and 6 PCGs) involved in chloroplastic fractions were selected for qRT-PCR analysis (Table S1). The gene expression trend of qRT-PCR data was in accordance with RNA-seq data (Figure 6). Pearson correlation coefficient between TPM (Transcripts per Million) values and relative transcript abundance estimated from qRT-PCR was found to be significant (*R* = 0.91, *p*-value < 0.01), indicating the reliability of our RNA-seq data. 

## 4. Discussion

### 4.1. Genome-Wide Identification, Characterization of lncRNAs in Allotetraploid C. hytivus

The prevalence of polyploid plants demonstrates the adaptive advantages of polyploid in evolution [59,60,61]. *C. hytivus* (HHCC, 2n = 4x = 38) is the first synthetic crop plant in *Cucumis* with increased ploidy level and showing growth vigor and improved adaptability [58]. *C. hytivus* has become one of the most important resources for introgression of needed traits from wild relatives into cucumber (*Cucumis hystrix* Chakr., 2n = 2x = 24) [62,63,64,65]. Taking advantage of its small genome size and clear genetic background, the newly synthesized allotetraploid has been examined as a model for many studies of allopolyploidization, such as genomic changes [66,67,68,69,70,71], gene expression patterns [39,72], epigenetic modification [73], chromosomal behavior [67,74], and miRNA regulation [75]. Like miRNAs, long non-coding RNA (lncRNAs) are crucial “regulatory” ncRNAs in the post-transcriptional and transcriptional regulation in eukaryotes [76]. However, the role of lncRNAs in the formation of *C. hytivus* remains undiscovered.

The advent of next-generation sequencing technology and the availability of the newly sequenced *C. hytivus* genome provided unprecedented opportunities to examine the impact of this important process on lncRNAs expression patterns. In the present study, a total of 1593 lncRNA loci (2206 isoforms) were identified in *C. hytivus* based on stringent criteria. lncRNAs were also identified in other polyploid plants, example given bread wheat (44,698) [77], cotton (8514) [34], tobacco (7423) [78], and *Brassica napus* (549) [79]. The identification efficiency of lncRNAs may be affected by their reference genome, RNA sequencing methods, and different identification strategies. Because the full-length cDNA library was enriched from Oligo (dT) magnetic beads, these lncRNAs without poly(A) tails have not been identified in the present study. However, most of the lncRNAs (>84.5%) [34] are transcribed by RNA polymerase II (Pol II), which are always capped, polyadenylated, and frequently spliced, much like coding mRNAs [55]. In the present study, we focus on the RNA pol II transcribed nuclear lncRNAs. 

The distribution of lncRNA throughout the chromosome can provide clues about possible functions and mechanisms of action. These lncRNAs located near protein-coding genes may regulate the expression of its neighbors by *cis*-acting [55]. Other lncRNAs transcribed from centromeric regions may play roles in centromere maintenance and cellular division [80]. The activated lncRNAs in polyploid cotton genome are predominantly transcribed from demethylated TE regions, especially from long interspersed nuclear elements (LINEs) [34]. Centromeric regions of cucumber genome are enriched with transposable elements (TEs) and lack of protein-coding gene sites [81]. In *C. hytivus*, lncRNA locus display an even distribution across the chromosomes (Figure 2a), with active transcription from centromeric regions. Our results suggested that lncRNAs located near centromeres or in gene deserts are implicated to act distally or have other effects in genome duplication.

Furthermore, approximately 19,252 gene pairs were one-to-one syntenic in two subgenomes (23,108 genes in subH; 22,535 genes in subC) (Figure S2b). These highly syntenic genomes helped identify the homologous lncRNA loci. Sequences of the lncRNA pairs with positional similarity within *C. hytivus* genome were compared, which allowed identification of 145 pairs of homeologous loci (Figure S2a). Using the collinear method, 103 homologous lincRNA loci with sequence similarities were identified in soybean, a diploidized ancient tetraploid crop [82]. In general, the primary model for rapid lncRNA emergence and decay in plants whole genome duplications and genome rearrangements may result in the lncRNA non-conservativeness. Although lncRNAs show less conservation at the primary sequences compared with mRNAs, some lncRNAs show striking conservation at the level of synteny and function [83].

According to the location relative to the nearest protein-coding gene, lncRNA can be further grouped into sense, antisense, intronic, and intergenic lncRNA [11]. In most plants, lncRNAs that come from the intergenic regions constitute the highest proportion, such as 85.1% in tomato [84], 79.7% in *Brassica napus* [47], and about 90% in cotton [34]. Similar result was also observed in our study, the majority of lncRNAs were located in intergenic regions (44.6%). However, divergent results were also reported in some other plants. For example, antisense lncRNAs are abundant in *Arabidopsis thaliana* [30], sense lncRNAs have a higher proportion in *Populus tomentosa* (75.6%) [85] and *Ananas comosus* [86]. Therefore, the location of lncRNAs may vary significantly from one species to another.

Candidate transcripts with only one exon have been removed in this study, because transcripts with two exons or more are more stable than unspliced (contain single-exon) transcripts. Despite this, most of the *C. hytivus* lncRNAs are relatively shorter and contain fewer exons than the protein-coding genes, consistent with the results of studies on tomato [84], *P. tomentosa* [85], and cotton [34]. Meanwhile, the transcript levels of the lncRNAs were also significantly lower than those of the mRNAs. Similar results were observed for lncRNAs in other plant species, such as Arabidopsis [87], rice [88] and *P. tomentosa* [85]. *C. hytivus* as a nascent polyploid plant, its lncRNAs showed both conventional characteristics and new special aspects in polyploid. It is very likely that these lncRNAs enable *C. hytivus* with greater physiological and ecological plasticity. Thus, it is necessary to compare the expression levels of lncRNAs in different samples and explore their regulation roles in the allopolyploidization process. 

### 4.2. Non-Additive lncRNA Expression under Intergeneric Hybridization and Polyploidization

Polyploidy is a major driver of genome evolution in dicotyledons. Genome-wide changes including variations in mRNA expression and alteration of epigenetic modifications, such as DNA methylation and histone modification, are frequently observed in polyploids. Most PCGs in synthetic allopolyploid are expressed at mid-parent level [8,9]. In our study, the hybridization and subsequent genome duplication also resulted in many intermediate morphological traits in *C. hytivus*, including plant height and stem diameter (Table S7), which was consistent with our previous results [58]. By contrast, we found that lncRNA expression changed dramatically by the genomic shock of interspecific hybridization in hybrid; several lncRNAs are related to agronomic traits. The differently expressed lncRNAs in hybrid/allopolyploid compared with maternal parent (HH) were more than with paternal parent (CC), suggesting parental-biased expression under hybridization and allopolyploidization, which were consistent with PCGs [89,90,91]. However, contrary to PCGs, most differentially expressed (DE) lncRNAs showed up-regulation in the hybrid/allopolyploid relative to the parents. It implied that these DE lncRNAs had a potential regulatory role in polyploidization.

Based on the GO enrichment analyses of potential target gene (PTGs) of differently expressed lncRNAs, we found that many thylakoid membrane, energy metabolism, and photosynthesis related genes were differentially expressed in the allopolyploidization. Chlorophylls are of great importance in plant light-harvesting and play essential roles in responding to changes in environmental conditions [92]. Chloroplast, as the organelle for photosynthesis, its photosynthetic membrane is considered the most complex and ingenious structure in all biofilms. When the light and temperature conditions change, it will capture the light quantum and drive a series of redox reactions [93]. The primary reaction of photosynthesis occurs on thylakoid membrane in chloroplast, which contains photosynthetic pigments and electron transport chain components, and the conversion of light energy to chemical energy is carried out on thylakoids. [94]. Some lncRNAs of the wild banana may regulate thylakoid membrane to maintain photosynthesis in low temperature stress [95]. The biological metabolism of plant chlorophyll is a complex and precise process, which takes 15 steps and involves multiple genes such as glutamine tRNA synthetase gene (*glxt*) [96]. Recently, many lncRNAs related to chlorophyll synthesis have been found in *Ananas comosus* [86]. In previous studies, we found that the chloroplast development of allotetraploid *C. hytivus* was hindered, and the genes *HEMA1*, *HEME2,* and *POR* related to chlorophyll biosynthesis were inhibited, resulting in a decrease in chlorophyll content [57]. Meanwhile, the expression of lncRNA near the genes of chlorophyll-binding protein and heme-binding protein changed, which might further affect the lineage-specific emergence of photosynthesis. More subsequent experiments concerning the fluorescence in situ hybridization (RNA-FISH) and silencing of *C. hytivus* antisense lncRNAs chc06G011860-AS-1 etc. may provide more information for revealing the regulation mechanism of leaf yellowing mediated by allopolyploidization.

## 5. Conclusions

In this study, we identified 2206 lncRNAs in allotetraploid *C. hytivus* by high throughput sequencing, among which most lncRNAs (~66.1%) were found to be active in allopolyploidization. GO enrichments of genes that either overlap with or are immediate neighbors of these significantly differentially expressed lncRNAs compared with two ancestors and MPV suggested involvement of many biological processes, including chloroplast components and photosynthesis biological process. Moreover, investigations on photosynthetic parameters provide necessary information for possible molecular mechanisms that LINC-chc01G000070-1, chc01G006010-AS-1, chc05G021510-AS-1, chc06G011860-AS-1, and other lncRNAs may be the key epigenetic regulators of leaf chlorosis and photosynthetic enhancement in *C. hytivus*. These results present a comprehensive atlas of lncRNAs in *Cucumis* allopolyploidization and paves the way for future understanding of the function of lncRNAs in polyploidization.

## Supplementary Materials

The following are available online at https://www.mdpi.com/2073-4425/11/12/1500/s1. Figure S1: Materials used for RNA sequencing. Figure S2: Cumulative distribution function of coding probability of candidate lncRNA and mRNA against CPAT. Figure S3: Dot plot for lncRNA/gene collinear blocks on two subgenomes of allopolyploid *C. hytivus* (HHCC). Figure S4: Description of the sequence characteristics of lncRNAs in *C. hytivus* leaves. Figure S5: Gene expression correlations between three biological replicates of lncRNA and mRNA in *C. sativus* (CC), *C. hystrix* (HH), interspecific hybrid F_1_, allotetraploid *C. hytivus* (S_14_). Figure S6: Comparison of the expression levels of lncRNAs and protein coding transcripts. Figure S7: Volcano plot of differentially expressed lncRNAs. Figure S8: UpSetR plot of variants across eight differently expressed lncRNA set queries. Figure S9: The number of differentially expressed lncRNA genes detected in F_1_ hybrid and allotetraploid S_14_ progeny compared with diploid progenitors (MPV). Figure S10: GO enrichment analysis of PTGs of differently expressed lncRNAs in F_1_ hybrid compared with parents/MPV. Figure S11: Heat maps of lncRNAs and PTGs expression in F_1_ hybrid, allotetraploid S_14_ and their parents (HH, CC). Figure S12: Photosynthetic characterization of the three species about allotetraploid S_14_ and their parents (HH, CC). Table S1: Sequences of qRT-PCR primers. Table S2: The number of lncRNAs and gene overlapping with or without TEs. Table S3: Basic summary of the expression levels of lncRNAs and mRNAs. Table S4: Seven bins of MPV-F_1_-S_14_ expression profile of lncRNAs. Table S5: Annotation of potential target genes of lncRNAs involved in photosynthesis. Table S6: Effect of allotetraploidization on the expression of lncRNAs and their targets involved in photosynthesis. Table S7: Plant growth characteristics among the *C. hytivus* and diploid parents.
