# Supplementary material for: Global Profiling of lncRNAs Expression Responsive to Allopolyploidization in Cucumis
Source: Genes (Basel). 2020 Dec 12;11(12):1500. doi: 10.3390/genes11121500 (PMC7763881; doi:10.3390/genes11121500)
Supplement: Supplementary file 1 [file genes-11-01500-s001.pdf]

Supplementary Material:

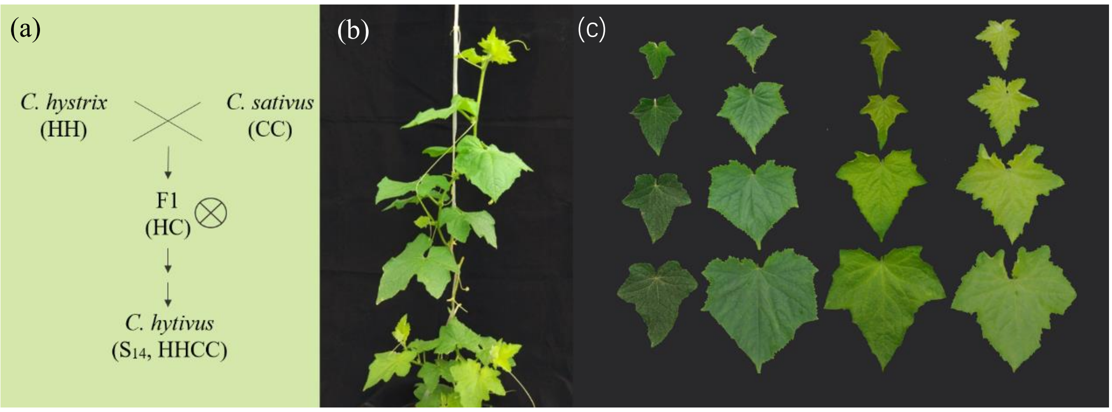

Figure S1. Materials used for RNA sequencing. **(a)** Schematic model for the diploid species (*Cucumis sativus*, CC; *Cucumis hystrix*, HH), F_1 hybrid, and the allotetraploid species (*C. hytivus*, HHCC) **(b)** Plant morphology of synthetic allotetraploid *C. hytivus* (S_{14}) **(c)** Leaf morphology (from 2st top leaf to 5th leaf) of the four species (from left to right): *C. hystrix*, *C. sativus*, F_1 and *C. hytivus*, photographed 30 days after germination.

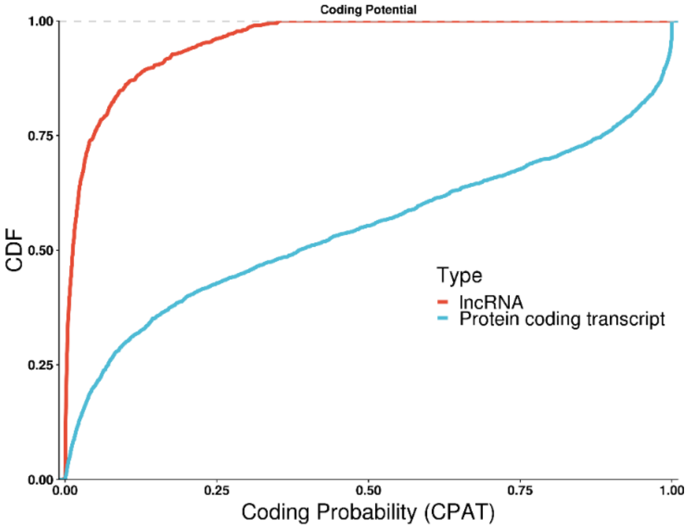

Figure S2. Cumulative distribution function of coding probability of candidate lncRNA and mRNA against CPAT.

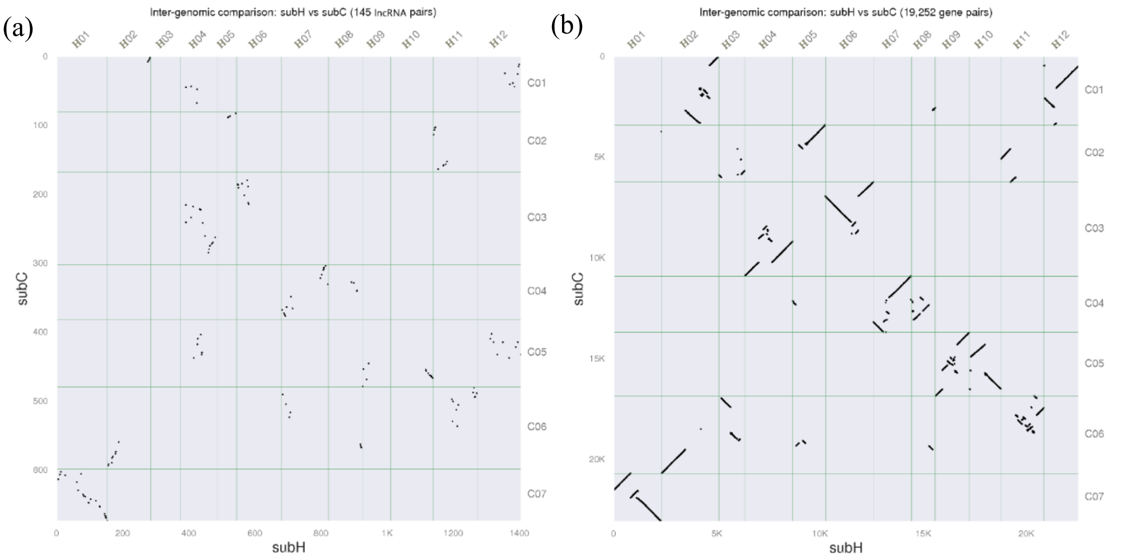

Figure S3. Dot plot for lncRNA/gene collinear blocks on two subgenomes of allopolyploid *C. hytivus* (HHCC). **(a)** lncRNA collinear blocks **(b)** protein coding gene collinear blocks. The result in PCGs showed that these chromosome C01—H02, H12; C02—H03, H05, H11; C03—H04, H06; C04—H07, H08; C05—H09, H10; C06—H02, H11, H03; C07—H01 have many syntenic loci. The result in lncRNAs showed that these chromosome C01—H02, H12, H04; C02—H05, H11; C03—H04, H06; C04—H07, H08; C05—H09, H10, H04, H12; C06—H02, H11, H07; C07—H01 have many syntenic loci. The collinearity of H03, H04, H07, H12 showed slight difference between lncRNA and PCGs. The syntenic lncRNA groups in remaining 8 HH chromosomes (66.7%) were consistent with genomic collinearity (PCGs).

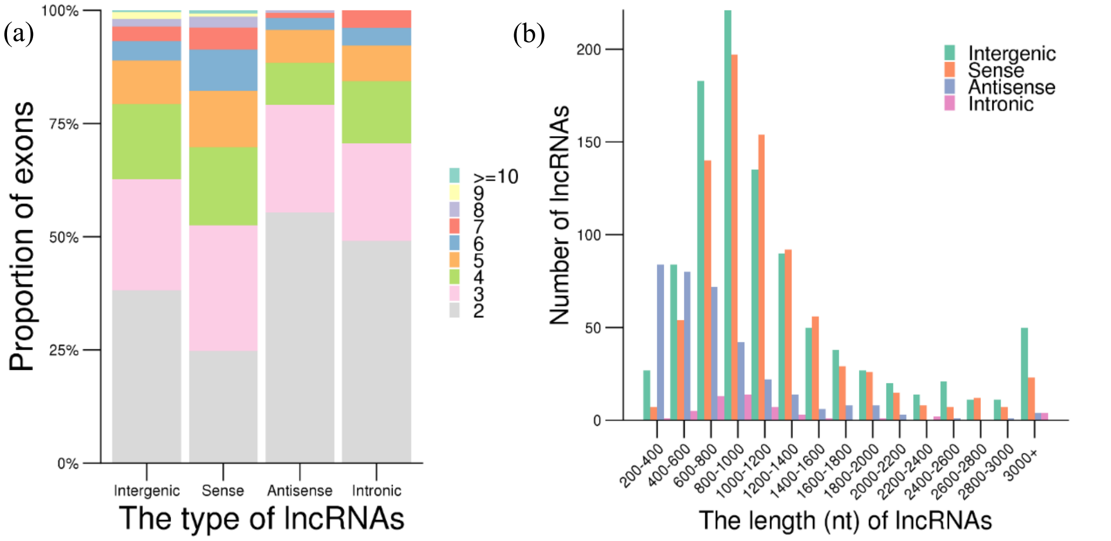

Figure S4. Description of the sequence characteristics of lncRNAs in *C. hytivus* leaves **(a)** Analysis of the exon numbers of each transcript of the four types of lncRNAs **(b)** Comparison of the transcript length distributions of the four types of lncRNAs.

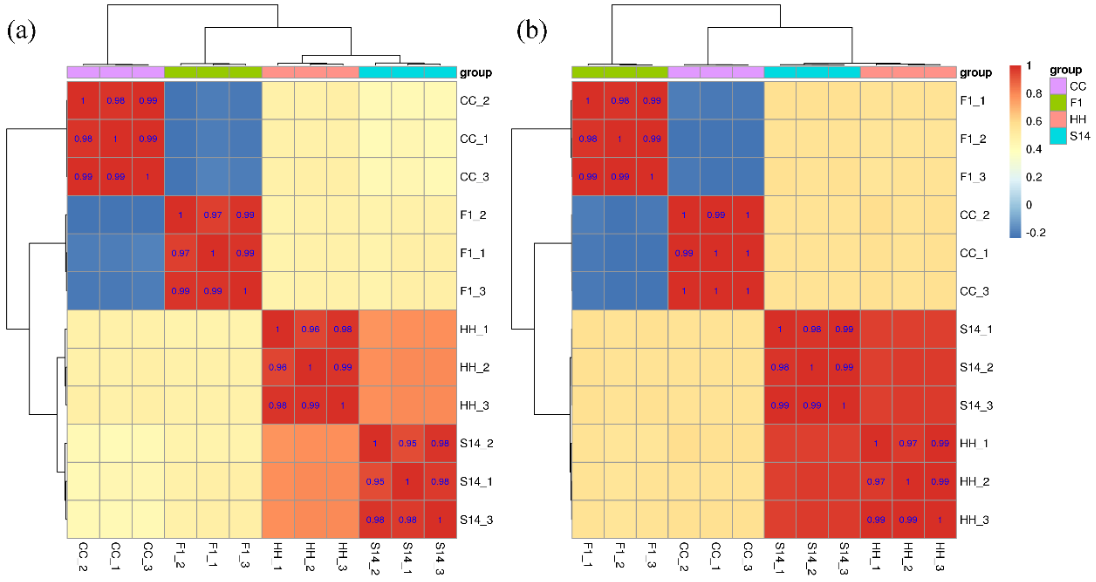

Figure S5. Gene expression correlations between three biological replicates of lncRNA **(a)** and mRNA **(b)** in *C. sativus* (CC), *C. hystrix* (HH), interspecific hybrid F_1, allotetraploid *C. hytivus* (S_{14}).

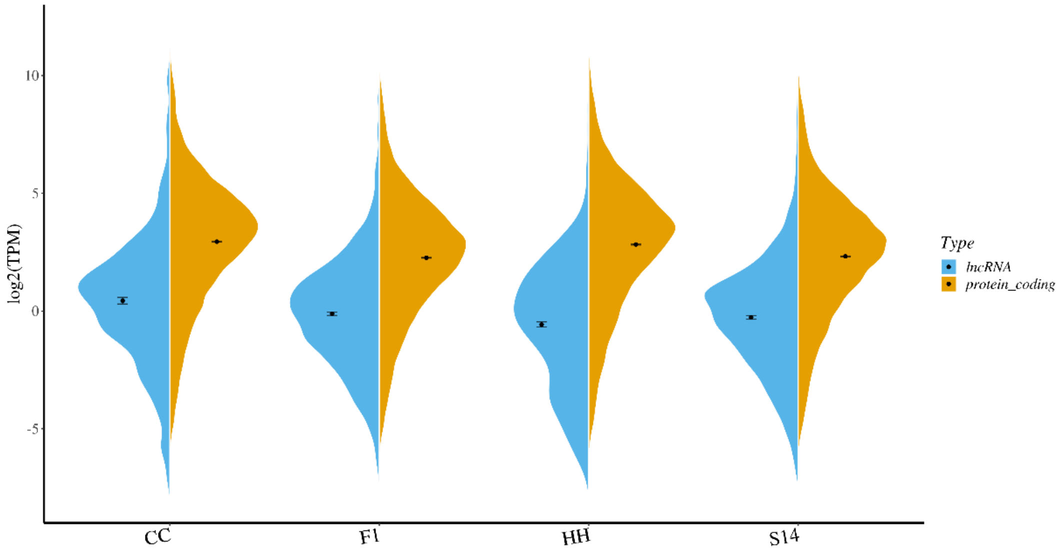

Figure S6. Comparison of the expression levels of lncRNAs and protein coding transcripts (TPM: Transcripts per Million).

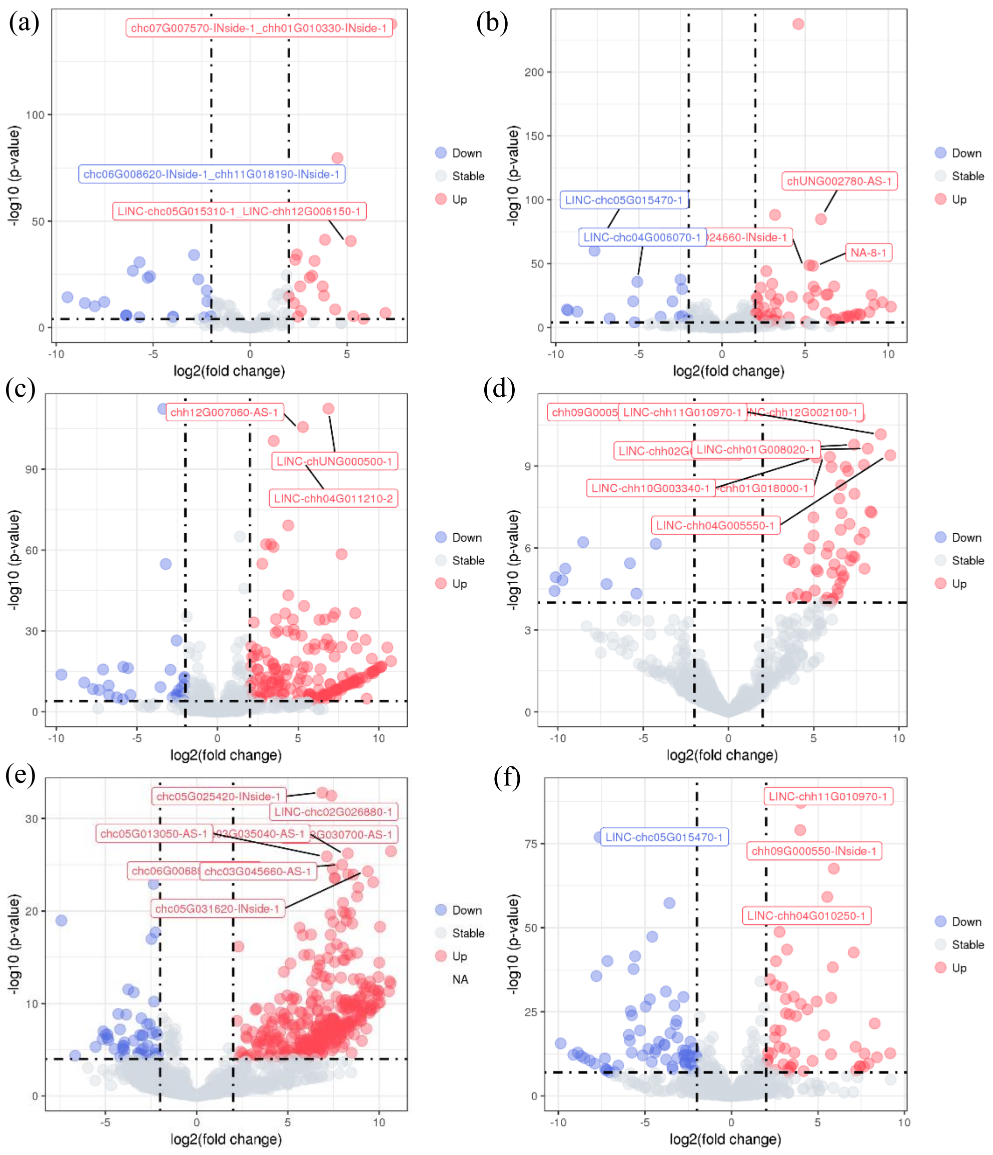

Figure S7. Volcano plot of differentially expressed lncRNAs between CC and HH **(a)**, between CC and S_{14} **(b)**, between HH and S_{14} **(c)**, between MPV and S_{14} **(d)**, between MPV and F_1 **(e)**, between F_1 and S_{14} **(f)**. Blue and red dots represent high expression lncRNA gene in the front and rear species, respectively.

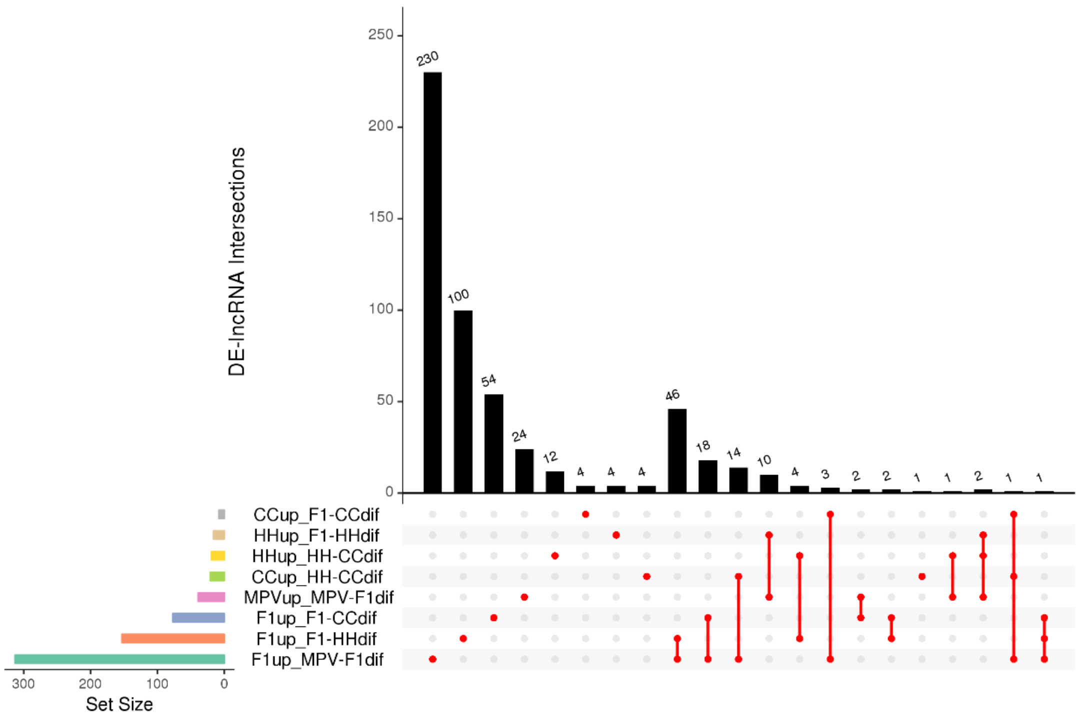

Figure S8. UpSetR plot of variants across eight differently expressed lncRNA set queries (F_1 hybrid and their diploid). For each set placed in the corresponding matrix cell, the red filled circle or light gray circle means that a set is or is not part of the given intersection. A vertical red line connects the topmost red circle with the bottommost red circle in each column to emphasize the column-based relationships. The size of the intersections is shown as a bar chart placed on top of the matrix. The bar chart shown to the left of the matrix represents the size of each set.

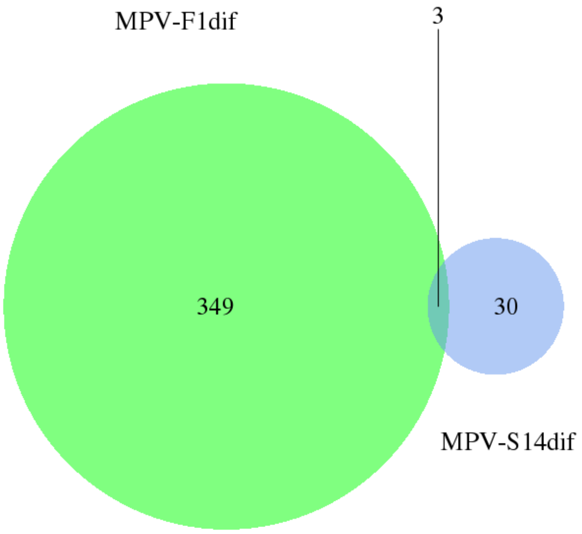

Figure S9. The number of differentially expressed lncRNA genes detected in F_1 hybrid and allotetraploid S_{14} progeny compared with diploid progenitors (MPV).

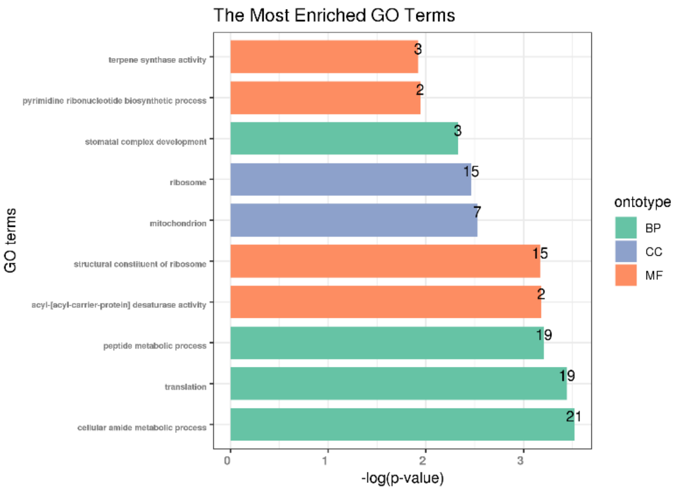

Figure S10. GO enrichment analysis of PTGs of differently expressed lncRNAs in F_1 hybrid compared with parents/MPV. Shown are significantly enriched GO terms (top10, P-value < 0.05). BP, biological process; MF, molecular function; CC, cellular component.

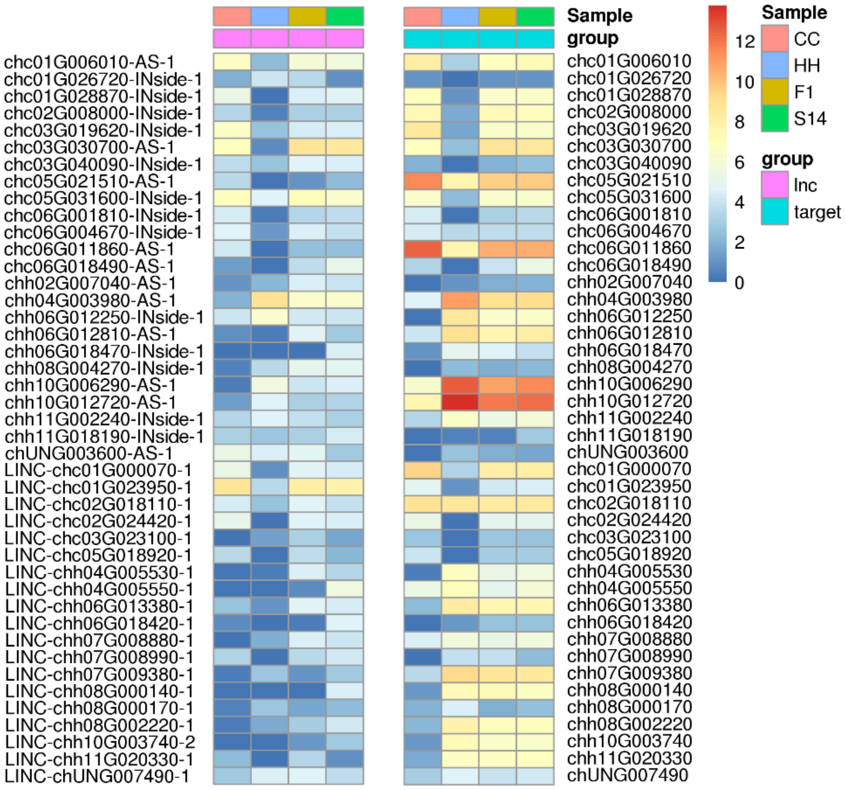

Figure S11. Heat maps of lncRNAs and PTGs expression in F_1 hybrid, allotetraploid S_{14} and their parents (HH, CC).

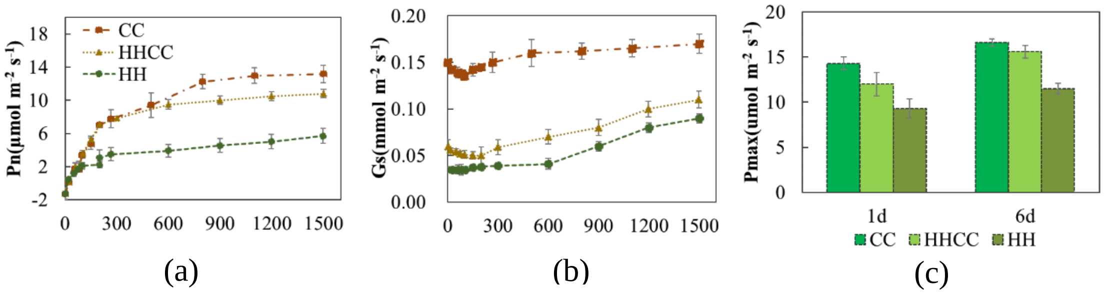

Figure S12. Photosynthetic characterization of the three species about allotetraploid S_{14} and their parents (HH, CC). **(a)** The net photosynthesis rates (Pn) of developing leaf in the three species. (Gs) in the three species. **(b)** Light response curve of stomatal conductance (Gs) in the three species. **(c)** The maximum net photosynthesis rate (Pmax) was measured at saturating light level in the fourth leaf from the tip of the stem, the second measurement was carried out after an interval of 5 days. The x-axis in a and b diagram represents 12 levels of leaf photo-synthetically active radiation (PAR). Vertical bars represent the mean values ± SE (n = 3).

Table S1. Sequences of qRT-PCR primers.

Seq ID	Type	Primer left sequence 5'→3'	Primer right sequence 5'→3'
chc03G019620-INside-1.1	lncRNA	AATTATAAGTTGGACAGTGT	ATTCTACACCATTTCATTAC
chc03G030700-AS-1.1	lncRNA	GAGCCTATAAATGACAGTAG	GAGAAAGATGGACACAAG
chc06G011860-AS-1.1	lncRNA	ATCTCGAGTGGATTACTT	GTACAAGCAATTGTAACTG
chc06G018490-AS-1.1	lncRNA	GAAATGTGAGATCTTAAACT	ATACTGCCTTTCCTATAAT
chh04G003980-AS-1.1	lncRNA	AAAGGATATTATGACGGTA	TCACTAATGATGTAAATGGT
chh06G012810-AS-1.1	lncRNA	CTCTCCAAGACTTTCTTC	GTGTTCATCTTCTCCTTC
LINC-chh08G002220-1.1	lncRNA	ACTCTTCTCCTTTCTCAG	ACACAGCATACTAACCAC
LINC-chh10G003740-2.1	lncRNA	GAGGTGAAAGTTTAATGTAT	TGAAACATATCACTACATCA
chc03G030700.1	mRNA	GAGAAAGATGGACACAAG	CTGGTATCATAACTTCATTG
chc06G011860.1	mRNA	GTGTTGTGAATGTTAACTAA	AATCTAAGGTGGATTTATAC
chh04G003980.1	mRNA	GGACGACGGTTCTCTGGCATTC	CGCATCAACTCCGCTCGGAATT
chh06G012810.1	mRNA	GGAGAAACAGATGTACATAT	GGAGAGAGAACATATGTAGA
chh08G002220.1	mRNA	AATATTTCTCAGATATCAGG	CAATGATCAATGTTATCAC
chh10G003740.1	mRNA	AAGAAGGAAGAGAAGAAG	AAACCTACCTGATATTGTT
F-box	reference	GGTTCATCTGGTGGTCTT	CTTTAAACGAACGGTCAGTCC

Table S2. The number of lncRNAs and gene overlapping with or without TEs.

	lncRNA	Gene
overlapping TE	1692(76.7%)	23962(51.2%)
without TE	514(23.3%)	22883(48.8%)

Table S3. Basic summary of the expression levels of lncRNAs and mRNAs.

**Type**	**Sample**	**log_2(TPM)**	**sd**	**se**
lncRNA	CC	0.4365	2.7565	0.0701
lncRNA	F_1	-0.1165	2.4401	0.0358
lncRNA	HH	-0.5743	2.7870	0.0548
lncRNA	S_{14}	-0.2721	2.5742	0.0382
protein coding	CC	2.9441	2.8028	0.0117
protein coding	F_1	2.2589	2.7164	0.0081
protein coding	HH	2.8240	2.8523	0.0121
protein coding	S_{14}	2.3213	2.6804	0.0080

Table S4. Seven bins of MPV-F_1-S_{14} expression profile of lncRNAs.

Profile ID	Number of genes	Expression patterns	
		MPV-F_1-S_{14}	
11	530	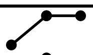	Up-Non
14	330	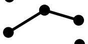	Up-Down
15	153	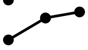	Up-Up
10	120	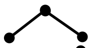	Up-Down
8	72	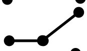	Non-Up
13	65	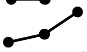	Up-Up
12	58	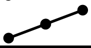	Up-Up

Table S5. Annotation of potential target genes of lncRNAs involved in photosynthesis.

**lncRNA**	**PTG**	**PTG Annotation**		
		**Swissprot**	**Nr**	**GO**
chc01G006010-AS-1	chc01G006010	--	uncharacterized protein LOC101204037	GO:0009535;GO:0009658;GO:0009773;GO:0015995;
chc01G026720-INside-1	chc01G026720	NADPH-dependent pterin aldehyde reductase	hypothetical protein	GO:0005829;GO:0009409;GO:0009507;
chc01G028870-INside-1	chc01G028870	3-oxoacyl-[acyl-carrier-protein] synthase II, chloroplastic	3-oxoacyl-[acyl-carrier-protein] synthase II, chloroplastic-like	--
chc02G008000-INside-1	chc02G008000	Ribonuclease E/G-like protein, chloroplastic	uncharacterized protein LOC101204095	GO:0003723;GO:0004540;GO:0006396;GO:0030246;
chc03G019620-INside-1	chc03G019620	Rhodanese-like domain-containing protein 14, chloroplastic	rhodanese-like domain-containing protein 14, chloroplastic-like	GO:0000023;GO:0007568;GO:0009535;GO:0009941;GO:0019252;
chc03G030700-AS-1	chc03G030700	Heme-binding-like protein At3g10130, chloroplastic	heme-binding-like protein At3g10130, chloroplastic-like	GO:0005634;GO:0006364;GO:0006399;GO:0009658;GO:0009793;GO:0010027;GO:0010228;GO:0016301;GO:0016310;GO:0016773;GO:0042644;GO:0042793;
chc03G040090-INside-1	chc03G040090	MEPFL3	EPIDERMAL PATTERNING FACTOR-like protein 3-like	--
chc05G021510-AS-1	chc05G021510	Chlorophyll a-b binding protein 7, chloroplastic	chlorophyll a-b binding protein 7, chloroplastic-like	GO:0009765;
chc05G031600-INside-1	chc05G031600	--	uncharacterized protein LOC101210271	--
chc06G001810-INside-1	chc06G001810	Anthranilate synthase alpha subunit 1, chloroplastic	hypothetical protein	GO:0000162;GO:0004049;
chc06G004670-INside-1	chc06G004670	MEPF2	protein EPIDERMAL PATTERNING FACTOR 2-like	--

chc06G011860-AS-1	chc06G011860	Chlorophyll a-b binding protein, chloroplastic	chlorophyll a-b binding protein of LHCII type 1	GO:0009765;
chc06G018490-AS-1	chc06G018490	Acyl-[acyl-carrier-protein] desaturase 6, chloroplastic	acyl-[acyl-carrier-protein] desaturase 6, chloroplastic-like	GO:0006631;GO:0006633;
chh02G007040-AS-1	chh02G007040	Cytochrome P450 CYP736A12	cytochrome P450 750A1-like	GO:0005506;GO:0016705;
chh04G003980-AS-1	chh04G003980	Plastocyanin, chloroplastic	plastocyanin, chloroplastic-like	GO:0005507;
chh06G012250-INside-1	chh06G012250	Rhodanese-like domain-containing protein 14, chloroplastic	rhodanese-like domain-containing protein 14, chloroplastic-like	GO:0000023;GO:0007568;GO:0009535;GO:0009941;GO:0019252;
chh06G012810-AS-1	chh06G012810	Glutamate synthase [NADH], amyloplastic	hypothetical protein	GO:0003824;GO:0005506;GO:0006537;GO:0006807;GO:0010181;GO:0015930;GO:0016040;GO:0016491;
chh06G018470-INside-1	chh06G018470	Sulfate transporter 4.1, chloroplastic	sulfate transporter 4.1, chloroplastic-like	GO:0008271;GO:0008272;GO:0015116;GO:0016020;GO:0016021;
chh08G004270-INside-1	chh08G004270	Putative cyclic nucleotide-gated ion channel 19	probable cyclic nucleotide-gated ion channel 20, chloroplastic isoform X3	--
chh10G006290-AS-1	chh10G006290	Ribulose bisphosphate carboxylase/oxygenase activase, chloroplastic	ribulose bisphosphate carboxylase/oxygenase activase 1, chloroplastic-like	--
chh10G012720-AS-1	chh10G012720	Ribulose bisphosphate carboxylase small chain, chloroplastic	ribulose bisphosphate carboxylase small chain, chloroplastic-like	GO:0004497;GO:0005618;GO:0009573;GO:0009579;GO:0009637;GO:0009853;GO:0009941;GO:0010114;GO:0010218;GO:0016020;GO:0022626;
chh11G002240-INside-1	chh11G002240	--	nicotinate phosphoribosyltransferase-like	GO:0003824;GO:0004516;
chh11G018190-INside-1	chh11G018190	Glycine--tRNA ligase 2, chloroplastic/mitochondrial	glycine--tRNA ligase 2, chloroplastic/mitochondrial-like, partial	GO:0000166;GO:0005524;
chUNG003600-AS-1	chUNG003600	Protein TIC	hypothetical protein	--
LINC-chc01G000070-1	chc01G000070	Magnesium protoporphyrin IX methyltransferase, chloroplastic	magnesium-protoporphyrin O-methyltransferase-like	GO:0015995;
LINC-chc01G023950-1	chc01G023950	Protein CHUP1, chloroplastic	hypothetical protein	--
LINC-chc02G018110-1	chc02G018110	Acyl-[acyl-carrier-protein] desaturase, chloroplastic	hypothetical protein	GO:0006631;GO:0006633;
LINC-chc02G024420-1	chc02G024420	Uncharacterized aarF domain-containing protein kinase	hypothetical protein	GO:0004672;GO:0005524;
LINC-chc03G023100-1	chc03G023100	Indole-3-acetic acid-amido synthetase	indole-3-acetic acid-amido synthetase GH3.6-like	GO:0009507;GO:0009826;GO:0010252;
LINC-chc05G018920-1	chc05G018920	Respiratory burst oxidase homolog protein	hypothetical protein	GO:0004601;GO:0005509;GO:0016020;GO:0016491;
LINC-chh04G005530-1	chh04G005530	Cytochrome P450	cytochrome P450 82C4-like	GO:0005506;GO:0016705;
LINC-chh04G005550-1	chh04G005550	Cytochrome P450	cytochrome P450 82A3-like	GO:0005506;GO:0016705;
LINC-chh06G013380-1	chh06G013380	2-isopropylmalate synthase 2, chloroplastic	hypothetical protein	GO:0003824;GO:0009098;GO:0019752;
LINC-chh06G018420-1	chh06G018420	Cytochrome P450 CYP736A12	cytochrome P450 71A1-like	GO:0005506;GO:0016705;
LINC-chh07G008880-1	chh07G008880	--	uncharacterized protein LOC101215901	GO:0005886;
LINC-chh07G008990-1	chh07G008990	Putative cyclic nucleotide-gated ion channel	probable cyclic nucleotide-gated ion channel 20, chloroplastic isoform X3	GO:0005216;GO:0006811;GO:0016020;
LINC-chh07G009380-1	chh07G009380	50S ribosomal protein L29, chloroplastic	50S ribosomal protein L29, chloroplastic-like	GO:0003735;GO:0005840;
LINC-chh08G000140-1	chh08G000140	Pyrroline-5-carboxylate reductase	pyrroline-5-carboxylate reductase-like	--
LINC-chh08G000170-1	chh08G000170	PsbP-like protein 1, chloroplastic	psbP-like protein 1, chloroplastic	GO:0005509;GO:0009523;GO:0009654;GO:0015979;
LINC-chh08G002220-1	chh08G002220	Ultraviolet-B receptor	hypothetical protein	GO:0009881;
LINC-chh10G003740-2	chh10G003740	Protein indeterminate-domain 5, chloroplastic	uncharacterized protein LOC101219959	--
LINC-chh11G020330-1	chh11G020330	Transketolase-2, chloroplastic	transketolase, chloroplastic-like	GO:0003824;
LINC-chUNG007490-1	chUNG007490	30S ribosomal protein S14, chloroplastic	ribosomal protein S14	GO:0003735;GO:0005840;

--: no function annotated.

Table S6. Effect of allotetraploidization on expression of lncRNAs and their targets involved in photosynthesis.

lncRNA	PTG	Correlation coefficient		Different Expression log_2Foldchange (lncRNA)			Different Expression log_2Foldchange (PTG)		
		R	p-value	MPV-S_{14}	CC-S_{14}	HH-S_{14}	MPV-S_{14}	CC-S_{14}	HH-S_{14}
chc01G006010-AS-1	chc01G006010	0.91	3.45E-05	-1.62	-1.50	-	-2.33	-1.18	-
chc01G026720-INside-1	chc01G026720	-0.62	0.0307	-5.40	-2.04	-	-0.91	0.23	-
chc01G028870-INside-1	chc01G028870	0.98	4.86E-08	-1.05	-0.96	-	-1.50	-0.36	-
chc02G008000-INside-1	chc02G008000	0.95	3.53E-06	-0.75	-0.64	-	-1.68	-0.54	-
chc03G019620-INside-1	chc03G019620	0.99	6.94E-11	-3.28	-3.17	-	-3.86	-2.72	-
chc03G030700-AS-1	chc03G030700	0.99	2.65E-10	2.05	2.16	-	1.52	2.68	-
chc03G040090-INside-1	chc03G040090	0.81	0.0013	0.68	1.04	-	-0.34	0.79	-
chc05G021510-AS-1	chc05G021510	0.94	6.11E-06	-2.07	-2.01	-	-3.53	-2.36	-
chc05G031600-INside-1	chc05G031600	0.90	7.95E-05	-0.98	-0.78	-	-0.87	0.29	-
chc06G001810-INside-1	chc06G001810	0.92	2.24E-05	-1.26	-1.18	-	-2.28	-1.14	-
chc06G004670-INside-1	chc06G004670	0.77	0.0031	-1.51	-1.40	-	-2.40	-0.77	-
chc06G011860-AS-1	chc06G011860	0.99	1.83E-10	-2.91	-2.83	-	-3.85	-2.70	-
chc06G018490-AS-1	chc06G018490	0.92	2.87E-05	5.88	5.97	-	2.27	3.41	-
chh02G007040-AS-1	chh02G007040	0.77	0.0033	2.55	-	2.76	0.71	-	1.73
chh04G003980-AS-1	chh04G003980	0.99	2.02E-13	-3.41	-	-3.68	-3.38	-	-2.37
chh06G012250-INside-1	chh06G012250	0.99	2.52E-10	-3.43	-	-3.52	-4.01	-	-3.00
chh06G012810-AS-1	chh06G012810	-0.29	0.354896	3.07	-	5.34	-2.43	-	-1.41
chh06G018470-INside-1	chh06G018470	0.99	4.57E-11	7.98	-	8.93	-2.82	-	-1.80
chh08G004270-INside-1	chh08G004270	0.30	0.3495	2.33	-	2.12	-1.20	-	-0.18
chh10G006290-AS-1	chh10G006290	0.99	2.95E-09	-1.21	-	-1.47	-2.41	-	-1.40
chh10G012720-AS-1	chh10G012720	0.99	2.39E-09	-1.94	-	-2.15	-3.22	-	-2.21
chh11G002240-INside-1	chh11G002240	0.81	0.0015	-2.57	-	-2.37	-1.69	-	-0.62
chh11G018190-INside-1	chh11G018190	0.88	1.39E-04	1.01	-	2.35	3.58	-	4.71
chUNG003600-AS-1	chUNG003600	0.56	0.0563	-4.26	-3.75	-2.52	-2.37	4.78	-1.36
LINC-chc01G000070-1	chc01G000070	0.97	3.41E-07	-1.72	-1.63	-	-2.74	-1.60	-
LINC-chc01G023950-1	chc01G023950	0.88	1.80E-04	-1.68	-1.58	-	-1.58	-0.42	-
LINC-chc02G018110-1	chc02G018110	0.18	0.5841	-1.31	-1.06	-	-2.38	-0.36	-
LINC-chc02G024420-1	chc02G024420	0.95	1.41E-06	-1.19	-1.10	-	-1.39	-0.25	-
LINC-chc03G023100-1	chc03G023100	0.32	0.3149	0.38	4.42	-	-0.97	0.16	-
LINC-chc05G018920-1	chc05G018920	0.65	0.0209	-2.33	-2.24	-	-2.31	-1.17	-
LINC-chh04G005530-1	chh04G005530	-0.15	0.6371	5.78	-	6.26	-2.17	-	-1.16
LINC-chh04G005550-1	chh04G005550	-0.14	0.6577	9.49	-	10.45	-2.55	-	-1.28
LINC-chh06G013380-1	chh06G013380	-0.08	0.8032	2.27	-	5.24	-2.01	-	-1.00
LINC-chh06G018420-1	chh06G018420	0.45	0.1422	6.65	-	9.10	1.41	-	2.43
LINC-chh07G008880-1	chh07G008880	0.10	0.7462	3.63	-	3.42	-1.33	-	0.04
LINC-chh07G008990-1	chh07G008990	-0.44	0.1551	1.20	-	8.45	-3.16	-	-2.15
LINC-chh07G009380-1	chh07G009380	0.79	0.0022	0.45	-	0.25	-2.18	-	-1.16
LINC-chh08G000140-1	chh08G000140	-0.09	0.7812	7.95	-	8.90	-1.64	-	-0.63
LINC-chh08G000170-1	chh08G000170	0.71	0.0102	-0.53	-	-0.68	-4.04	-	-2.93
LINC-chh08G002220-1	chh08G002220	0.35	0.2706	3.92	-	3.86	-2.16	-	-1.14
LINC-chh10G003740-2	chh10G003740	-0.09	0.7875	5.34	-	6.30	-2.07	-	-1.06
LINC-chh11G020330-1	chh11G020330	0.18	0.5696	-2.83	-	-	-0.68	-	0.35
LINC-chUNG007490-1	chUNG007490	0.65	0.0211	-1.39	1.05	-1.33	-1.65	2.14	-0.39

R: Pearson correlation coefficient between log_2(TPM) of lncRNA and PTG (potential target gene).

Table S7. Plant growth characteristics among the *C. hytivus* and diploid parents.

Growth period	Genotype	Plant height, cm	Stem diameter, mm	Leaf length, cm	Leaf width, cm
seedlings with three true leaves	*C. sativus*	33.23±0.15a	5.47±0.07a	6.9±0.26bc	7.2±0.88b
	C. ×hytivus	21.17±1.17b	5.2±0.05ab	9.13±0.83a	12.6±1.5a
	C.hystrix	14.57±1.00c	2.77±0.32d	5.8±0.64c	6.93±0.77b
	Species effect	**	**	**	**
after transferring 5 days	*C. sativus*	43.37±0.06a	6.07±0.03a	10±0.5a	12.07±0.38bc
	C. ×hytivus	28.23±0.06b	5.52±0.03b	10.27±0.5a	15.9±0.85a
	C. hystrix	20.17±0.032c	3.61±0.14c	6.6±0.7b	8.33±0.79d
	Species effect	**	**	**	**

Values are mean ± SE (n = 3). Different letters within the same column indicate significant differences at 0.05 ($P < 0.05$). ** represents significance $P < 0.01$.
